# One-year oral health outcome of a community-based trial in schoolchildren aged 6–7 years old in Tehran, Iran

**DOI:** 10.1371/journal.pone.0284366

**Published:** 2023-04-21

**Authors:** Azadeh Babaei, Afsaneh Pakdaman, Ahmad Reza Shamshiri, Pegah Khazaei, Hossein Hessari

**Affiliations:** 1 Community Oral Health Department, School of Dentistry, Alborz University of Medical Sciences, Karaj, Iran; 2 Research Centre for Caries Prevention, Dentistry Research Institute, Tehran University of Medical Sciences, Tehran, Iran; 3 Community Oral Health Department, School of Dentistry, Tehran University of Medical Sciences, Tehran, Iran; International Medical University, MALAYSIA

## Abstract

Promoting schoolchildren’s oral health is important, particularly in developing countries. This study was conducted to monitor the oral health of schoolchildren aged 6–7 years old following the implementation of an oral health promotion program in Tehran, Iran. The protocol was registered in the Iranian Registry of Clinical Trials (Code: IRCT20090307001749N4). A cluster random sampling method was applied, and the schools were randomly allocated to intervention and control groups. An intervention package consisting of a one-day workshop for parents and supervised toothbrushing for children was employed. In both groups, the Caries Assessment Spectrum and Treatment (CAST) and Oral Hygiene Index Simplified (OHI-S) were evaluated at baseline and at one-year follow-up in addition to the questionnaire data. Clinical data were collected by calibrated examiners at both intervals (Kappa = 89.8%, 87.68%) and analyzed using the SPSS software ver. 22.0. Of 739 children included at baseline, 593 were re-examined after one year (response rate = 74%). According to the Generalized Estimating Equation (GEE) analysis, considering the confounding effect of time, significantly more children in the control group had deciduous molars with a score of 3 and higher compared to the intervention group (OR = 1.79; 95% CI:1.17–2.73, p = 0.007). The oral hygiene status of the children significantly improved in the intervention group compared to the controls (B = -0.27; 95% CI: -0.45 –-0.08, p = 0.005). After one year, the improvement in the oral health-related attitude of parents and children’s oral health behavior was marginally significant in the intervention group compared to the control group [0.2 (0.17) vs. -0.13 (0.05), p = 0.096] and [0.06 (0.06) vs. -0.05 (0.04), p = 0.09], respectively. However, the impact on the oral health-related knowledge and self-reported behavior of the parents was not significant. In the intervention group, children had less caries and a better oral hygiene status compared to the controls after one year.

## Introduction

Dental caries influences the social life and well-being of people and has a great impact on the quality of life. In children, untreated caries has more negative impacts as it influences social life, school attendance, speech, and self-esteem [1, 2]. Oral health improvement is recommended through community programs, especially in school-aged children. It is necessary to promote awareness of caries risk factors, especially in developing countries. Supervised toothbrushing programs in the school setting has been well-documented as an effective method to improve the oral health of children [1, 3].

Schools are considered as an important setting to transfer information to children and their families as they offer an efficient and effective way of reaching over one billion children worldwide [1]. The school setting makes it possible to detect oral health problems at early stages and deliver care [4]. Many studies, including a review by Cooper et al., recommend brushing in school [5]. In addition, a large body of evidence demonstrates the effectiveness of fluoride delivered in various forms such as varnish, gel, toothpaste, etc. to prevent dental caries [6, 7]. The guidelines recommend that supervised toothbrushing should start from approximately 6 months of age up to the age of 7 [8].

A key component of supervised toothbrushing programs is ensuring plaque removal effectively via brushing twice daily using fluoride toothpaste under the supervision of a parent/caregiver [8]. The supervised toothbrushing technique is highly recommended at the time of eruption of permanent first molars at about six years of age. Most parents may not be aware of tooth eruption and neglect the plaque removal [9]. In this regard, the bucco-lingual technique is considered an effective method in plaque removal, especially when supervised by parents [10].

There are common indices to measure dental caries in the community-based surveys such as the DMFT/dmft index [11] although this index does not reflect the consequences of untreated dental caries, including pain and infection. Due to limitations of the newly developed indices such as the ICDAS [12] and PUFA/pufa [13], a new index was developed by Frencken et al. in 2011 [14] to measure a wide range of caries from non-cavitated to advanced cavitated lesions as well as the clinical consequences of caries. Considering the new concept of caries management, it is important to record a wider range of caries from non-cavitated to cavitated lesions. This is more important in developing countries where service delivery is limited and caries mainly remains untreated in children [15].

According to a national survey in 2012, the prevalence of tooth decay is high in Iranian children. The mean dmft/DMFT index was 5.16 (SD = 0.38) in 6-year-old children, and only 13% of children were caries-free in this age group [15]. There is also evidence of a wide spectrum of dental caries in Iranian children [16], indicating the importance of the management of non-cavitated lesions as well as service delivery to the high-risk children. Since no study has evaluated the oral health status of Iranian children, especially following oral health promotion programs using detailed indices, the purpose of the present study was to evaluate the impact of an oral health promotion program, including supervised toothbrushing, on the oral health status of schoolchildren aged 6–7 years old in Tehran, Iran.

## Materials and methods

This study was a parallel-arm, examiner/statistician-blind, one-year follow-up of a community-based randomized controlled trial of an oral health promotion program including oral health education and supervised toothbrushing. This study was conducted on schoolchildren aged 6–7 years old in 19 educational districts of Tehran, Iran.

### Ethical considerations

The Ethical clearance was obtained from the Ethics Committee of Tehran University of Medical Sciences, Tehran, Iran (IR.TUMS.REC.1394.1730). The study was also registered in the Iranian Registry of Clinical Trials (Code: IRCT20090307001749N4). Informed consent was obtained from parents (mothers) after explaining the outline of the study.

### Sampling

**Fig 1:**
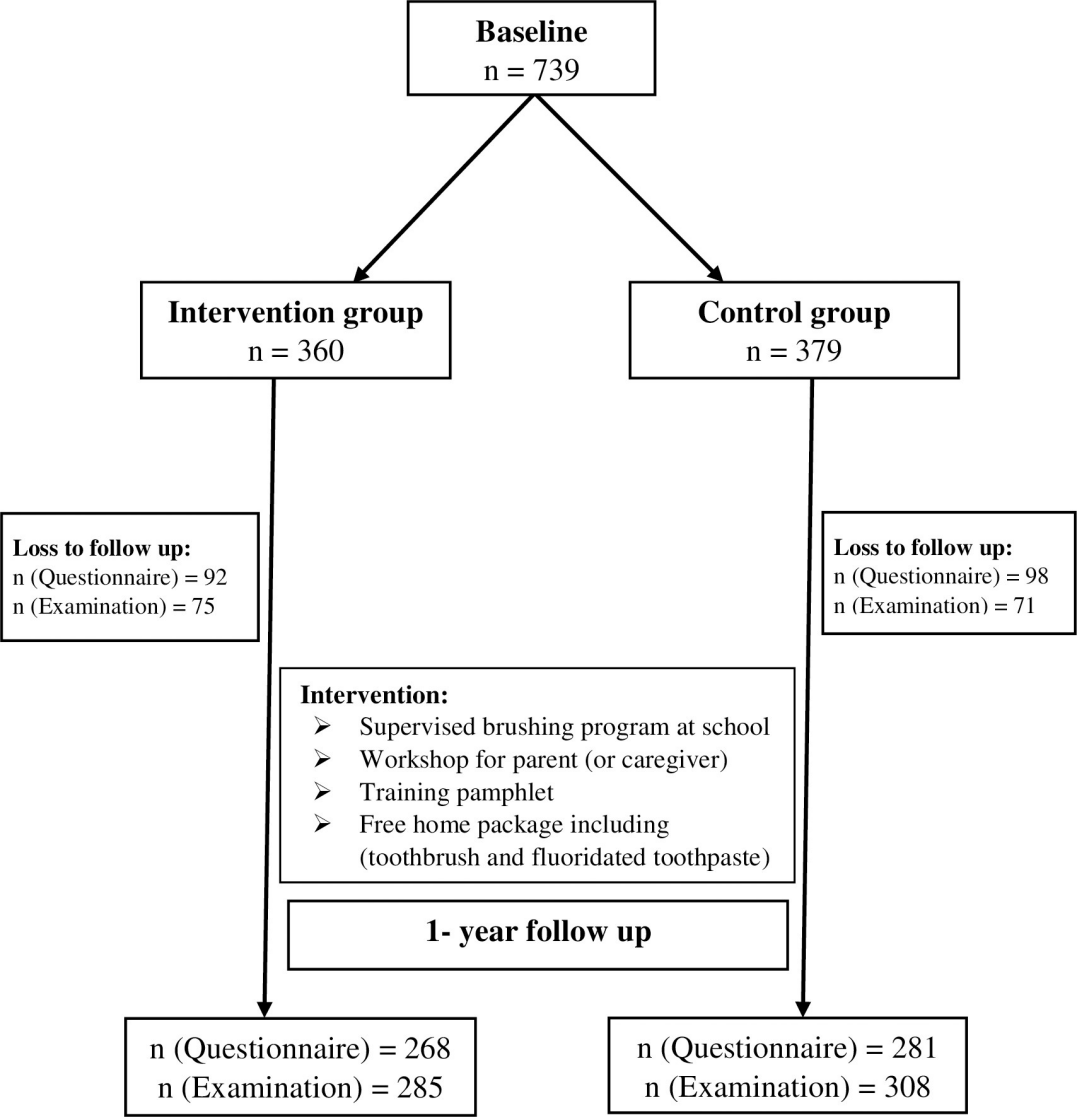
Overview of the study at baseline and one-year follow-up according to the CONSORT flow diagram.

This study was a one-year follow-up of a previously described community trial of school children from February of 2016 to March 2017 (methodology explained elsewhere) [17]. After considering schools as clusters, randomization applied at the class level, and one class was selected randomly from each school. Thus, 12 schools/classes were selected in each arm of the study. Blinding was done and the participants, the statistician, and the examiner (PKH) were not aware of the allocated group [16]. We calculated the sample size using the formula based on comparison of two proportions in the case and control groups. This proportion was the percentage of children with pulp involvement calculated as 0.3 based on the pilot study of 20 students. Accordingly, the sample size was calculated 720 subjects, 360 subjects in each group, with a power of 80%, α = 0.05, β = 0.2, loss to follow-up of 20%, and design effect of 1.5. The framework of the study is presented in (Fig 1). The inclusion criteria were schoolchildren aged 6–7 years old that went to public schools and their parents gave informed consent. In addition, the children that had allergies to fluoride, physical disability, systemic diseases, or dental emergencies were excluded.

### Intervention

#### Theoretical session

**Fig 2:**
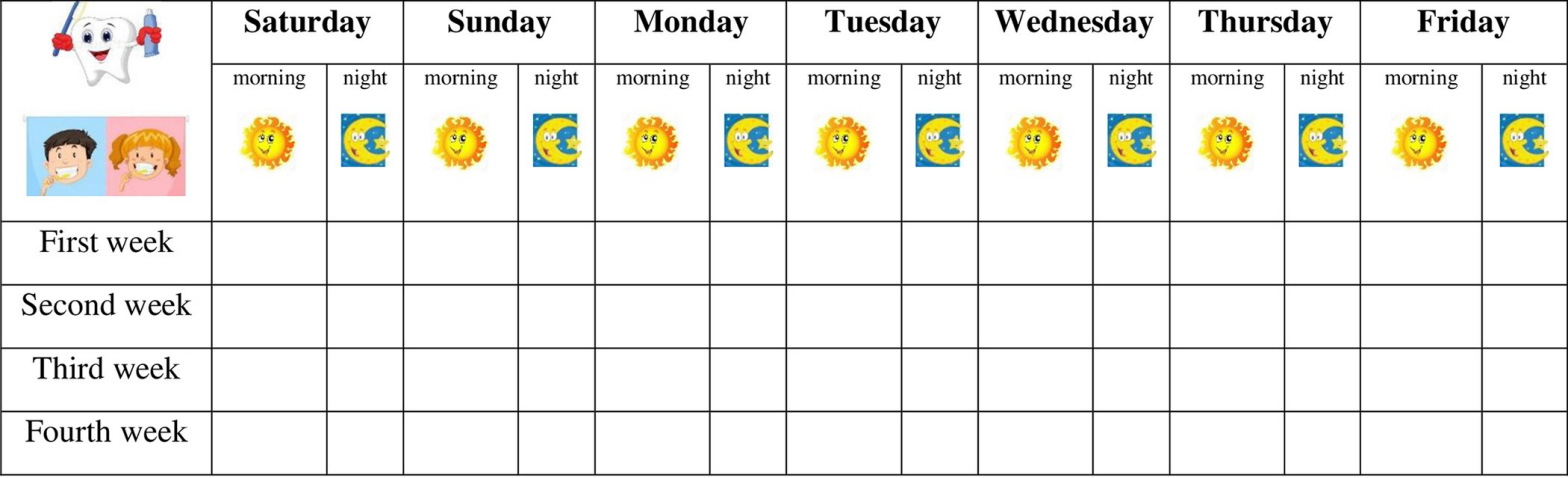
Weekly guide of supervised toothbrushing for children for use at home.

In the intervention group, the parents/caregivers of the children were invited to participate in a one-day workshop on supervised toothbrushing according to the World Health Organization (WHO) format [18] that was delivered to parents using a PowerPoint presentation. In addition, due to the importance of the permanent first molars, the professional buccolingual technique was presented using a video clip followed by a question and answer session. A free-of-charge package containing a toothbrush and fluoride toothpaste (1000 ppm) and a weekly guide was also given to the parents for use at home (Fig 2).

The content of the parents’ workshop included the order of the eruption of deciduous and permanent teeth, the position of the first permanent molar teeth, the concept of dental plaque and dental caries, toothbrushing technique (particularly the buccolingual technique for the first permanent molars), the importance of supervised toothbrushing, proper nutrition, and fluoride and fissure sealant therapy. Finally, a pamphlet and a parent supervision package were given to the parents. In addition, an overview of the etiologic factors of tooth decay was presented to children in the classroom including the importance of toothbrushing, proper brushing technique, fluoride use, and sugar consumption. The educational intervention package for children and parents was repeated twice in the period of one-year after the initial examination.

#### Practical session

In the intervention group, after coordinating with the teaching staff, the students were taught how to brush properly including the first permanent molars under the supervision of the main researchers (AB, AP) in the school setting in groups of 6–8 children. A free toothbrush with a pea-sized amount of fluoride toothpaste was given to each student. It is noteworthy that all children in both intervention and control groups received varnish fluoride (22,600 ppm) biannually as part of the national fluoride program offered by the Ministry of Health and Medical Education (MOHME).

The examiners were calibrated, and dental caries was measured according to the CAST index [14] by the first examiner (AB) calibrated by an expert (AP) (inter-examiner kappa: 89.8, intra-examiner kappa: 91.2). In addition, one year after the intervention, the examination was repeated by another dentist (PKH) that was blind to groups and was calibrated by an examiner (AB) (Kappa = 87.68%).

#### Questionnaire

A previously validated questionnaire [19] was used to assess oral health related knowledge, attitude, and self-reported behavior of parents. The questionnaire had three sections, including: A) parents’ knowledge of etiologic factors of dental caries, cariogenic diet, importance of brushing and its frequency, and using fluoride (9 questions). B) parents’ attitude about the importance of dental caries and its prevention (6 questions), and C (a) parents’ self-reported behavior including the frequency of brushing, using fluoride toothpaste, and consuming sugary snacks and drinks (3 questions) and C (b) children’s oral health related behavior including brushing frequency, using fluoride toothpaste, and having sugary snacks and drinks (3 questions).

In the knowledge section, correct answers scored one and incorrect answers scored zero. In a Likert scale, correct answers included “I strongly agree” and “I agree” for questions with a positive answer and “I disagree” and “I strongly disagree” for questions with a negative answer. Then, the sum score of the questions (0 to 9) was calculated for each subject. In the attitude section, a 5-point Likert scale from “I strongly agree” to “I strongly disagree” was dichotomized and the total score was calculated (6 to 30). In the self-reported behavior section, the sum score of questions (0 to 6) was calculated for each person. Parents’ and children’ behavior questions included three questions. The correct answers scored 1 vs. 0 for incorrect answers. Then, the sum score (0 to 3) was calculated for each person.

### Statistical analysis

All data from clinical examination and questionnaires were analyzed using the SPSS version 22.0 released 2013 (IBM SPSS Statistics for Windows, Armonk, NY: IBM Corp). Descriptive statistics were used for reporting demographic variables. In the control and intervention groups, the Oral Hygiene Index Simplified (OHI-S) and Caries Assessment Spectrum and Treatment (CAST) were evaluated at baseline and one-year follow-up. The questionnaire data including oral health related knowledge, attitude and self-reported behavior were analyzed. The Generalized Estimating Equation (GEE) with exchangeable correlation matrix was used to consider the cluster effect of school and time. In this analysis, the school code was considered as a “subject variable” and the student code and time were considered as the “within-subject variables”. In the GEE analysis, a linear model was used for quantitative outcomes (oral hygiene, knowledge, attitude, and self-reported behavior in relation to oral health), and a binary logistic model was used for the qualitative outcome (CAST index). In addition, in this analysis, time and intervention were considered as independent variables (predictors). The oral hygiene, caries, and oral health related knowledge, attitude and self-reported behavior considered as outcome variables. The models were examined considering the intervention × time interaction. The significance level was set at 0.05.

## Results

In the community trial, 739 children aged 6–7 years were included at baseline of whom 593 were re-examined after one year (response rate = 74%). In addition to clinical data, 549 children returned the completed questionnaire on oral health-related knowledge, attitude, and self- reported behavior of parents (Table 1).

**Table 1:** Demographic characteristics of study participants in control and intervention groups in one-year follow-up.

		Control		Intervention	
		N	(%)	N	(%)
Gender (n = 593)					
	Boy	141	45.8	136	47.8
	Girl	167	54.2	149	52.2
	Total	308	100	285	100
Father’s Education (n = 534)					
	Illiterate/Elementary school^1^/Middle school^2^	47	17.3	46	17.5
	Unfinished high school education or high school diploma^3^	96	35.5	98	37.3
	Associate^4^/Bachelor’s/Master’s degree/Doctorate	128	47.2	119	45.2
	Total	271	100	263	100
Mother’s Education (n = 538)					
	Illiterate/Elementary school^1^/Middle school^2^	38	13.8	35	13.3
	Unfinished high school education or high school diploma^3^	114	41.5	119	45.3
	Associate^4^/Bachelor’s/Master’s degree/Doctorate	123	44.7	109	41.5
	Total	275	100	263	100
Childbirth order (n = 494)					
	First	166	65.1	162	67.8
	Second	74	29	64	26.8
	Third and more	15	5.9	13	5.4
	Total	255	100	239	100
Number of children in the household (n = 509)					
	One child	88	34.5	91	35.8
	Two children	139	54.5	137	53.9
	Three children and more	28	11	26	10.3
	Total	255	100	254	100
Socio-economic status^5^ (n = 435)					
	Less than 20	99	45.4	108	49.8
	Equal or more than 20	119	54.6	109	50.2
	Total	218	100	217	100

^1^5 years

^2^6–8 years

^3^ 9–12 years

^4^ 2 years of academic education

^5^ According to housing area (m^2^ per person)

### Demographic characteristics

Table 1 presents the socio-demographic characteristics of the participants in the intervention and control groups including gender, father’s education level, mother’s education level, childbirth order, number of children in the household, and socioeconomic status at baseline and after one year. In the one-year follow-up, girls comprised 52.5% and 54.2% of the subjects in the intervention and control group, respectively. Regarding the parents’ education level, about half of the fathers and mothers in both groups had a university education (45.2% vs 47.2% and 45.3% vs 41.5, respectively). In terms of the socio-economic status, about half of the participants in both groups lived in houses with an area of equal to or more than 20 square meters per person (50.2% and 54.6% of the subjects in the intervention and control groups, respectively).

### Questionnaire

At baseline, the sum score of the parents’ oral health-related knowledge, attitude and behavior were compared as the (mean and standard deviation of the sum score) between the intervention and control groups (6.76±1.89 vs. 6.92±1.59, 5.04±1.33 vs. 5.31±1.05, and 2.59±0.64 vs. 2.60±0.62, respectively), indicating no significant difference. In addition, the children’ oral health behavior (mean and standard deviation of the sum score) was compared between the intervention and control groups (2.12±0.82 vs. 2.11±0.80). Table 2 shows changes in the mean score of the parents’ oral health related knowledge, attitude and self-reported behavior compared to baseline in the intervention and control groups after one year according to the GEE analysis. There was improvement in the intervention group compared to the control group; however, the changes in the parents’ attitude and children’s behavior were marginally significant.

**Table 2:** Evaluation of changes in the mean score of oral health knowledge, attitude, and practice in one-year follow-up compared to baseline in control and intervention groups.

	Control group		Intervention group		
	Mean	Std. Error	Mean	Std. Error	p-value*
Parents’ oral health knowledge	-0.04	0.11	0.3	0.25	0.21
Parents’ oral health attitude	-0.13	0.05	0.2	0.17	0.096
Parents’ oral health behavior	-0.09	0.05	-0.04	0.03	0.26
Child’s oral health behavior	-0.05	0.04	0.06	0.06	0.09

### Dental caries according to CAST index

**Fig 3:**
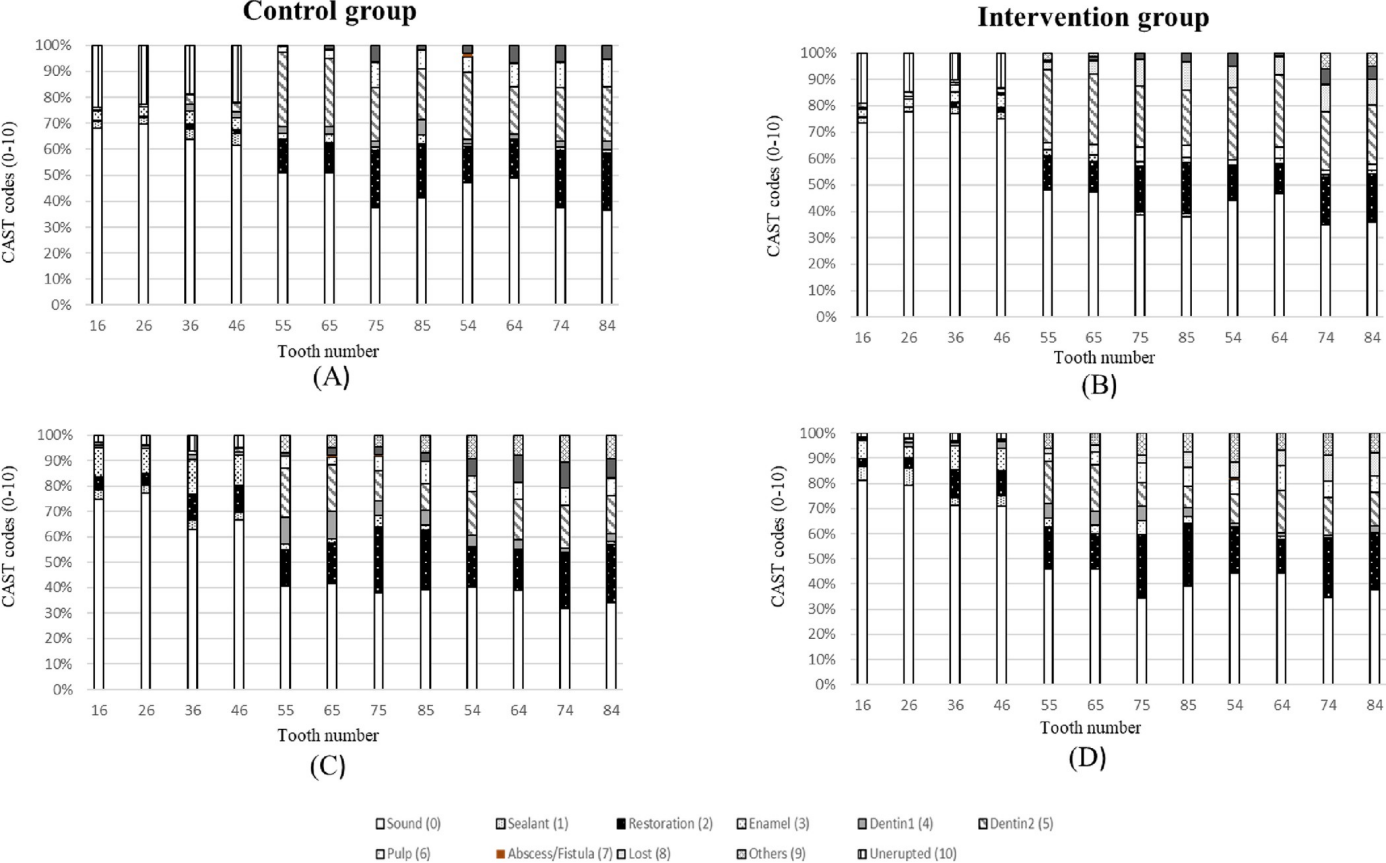
Caries status according to the CAST index in the primary and first permanent molars in the control and intervention groups at baseline (A and B) and 1-year follow-up (C and D).

Table 3 shows a CAST score ≥ in primary molar teeth in the intervention and control group at baseline compared to the one-year follow-up. According to the GEE analysis, after considering the confounding effect of time, significantly more children in the control group had deciduous molars with a score ≥ compared to the intervention group (OR = 1.79; 95% CI: 1.17–2.73, p = 0.007). The caries status of children in the control and intervention groups in primary and permanent dentition at baseline and one-year follow-up according to the CAST index demonstrated in Fig 3.

**Table 3:** Comparison of changes in caries status of primary molar teeth in one-year follow-up between intervention and control groups according to the CAST index.

			One-year follow-up	
			CAST < 3	CAST > = 3
Baseline	Control group	CAST < 3	46 (14.9%)	31 (10.1%) *
		CAST > = 3	15 (4.9%)	216 (70.1%)
	Intervention group	CAST < 3	35 (12.3%)	13 (4.5%) *
		CAST > = 3	27 (9.5%)	210 (73.7%)

* p = 0.007, Generalized Estimating Equation, adjusted for Time * Intervention/Control.

### Oral hygiene

**Fig 4:**
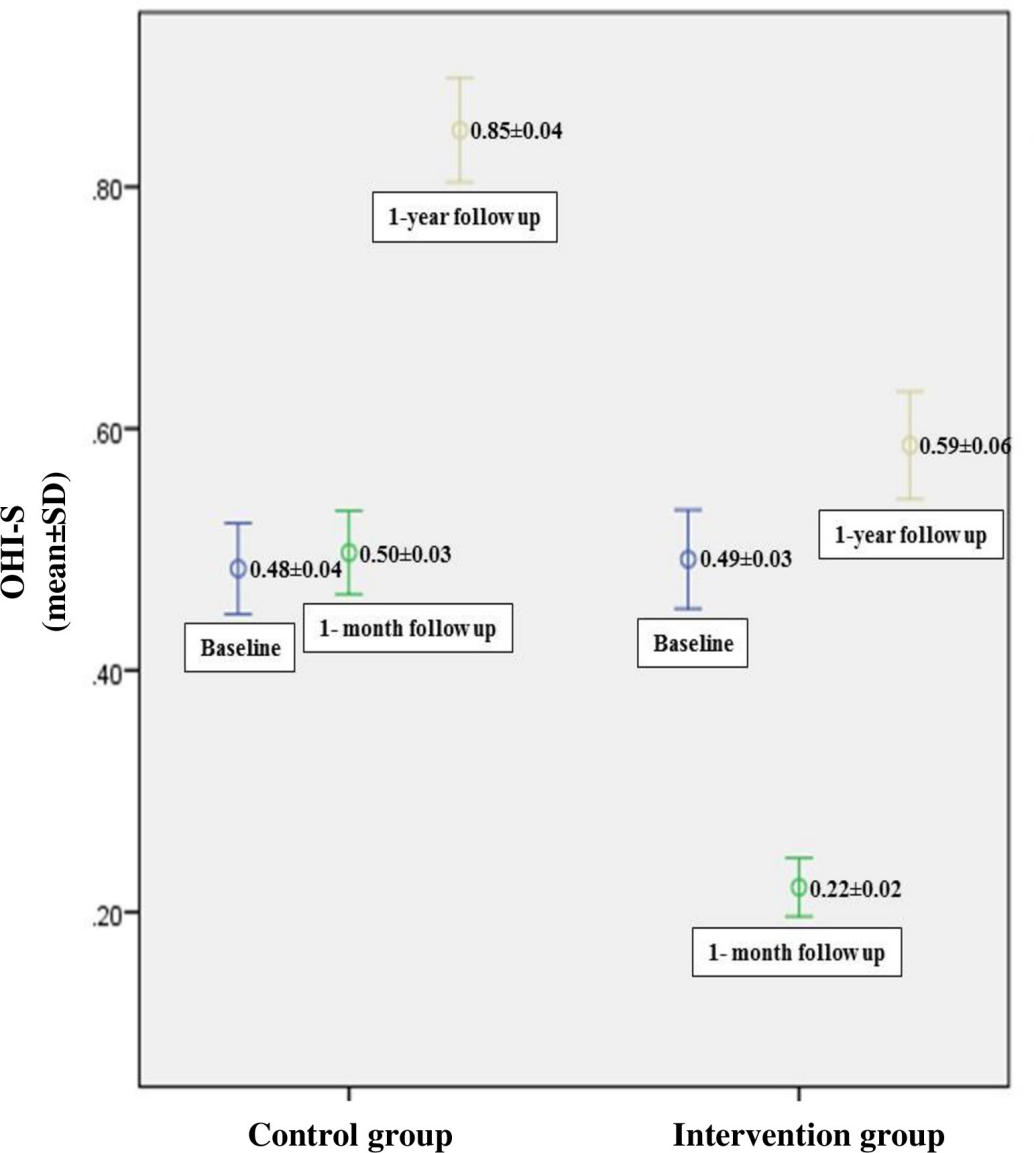
The mean OHI-S score in children at baseline, one-month follow-up, and one-year follow-up in intervention and control groups.

The oral hygiene status of the children measured according to the OHI-S index. At baseline, the mean and standard deviation of the oral hygiene score was 0.49±0.03 and 0.48±0.04 in the intervention and control group respectively, indicating no significant difference [16]. The changes (Δ) of the OHI-S score in the post-test evaluation in the one-month follow-up are reported elsewhere [17]. After one-year, the Δ OHI-S was 0.59±0.06 and 0.85±0.04 in the intervention and control group, respectively (p = 0.01). The comparison between baseline, one-month follow-up, and one-year follow-up of oral hygiene status measure according to Δ OHI-S presented in (Fig 4). The results of the GEE analysis, after adjustment for the confounding effect of time, showed a significantly higher OHI-S score in the control group compared to the intervention group (B = -0.27, 95% CI: -0.45 –-0.08, p = 0.005). Accordingly, the GEE analysis showed improved oral hygiene status in the one-year follow-up.

## Discussion

The present study evaluated the one-year impact of an oral health promotion program including parents’ education and supervised toothbrushing on oral health status of schoolchildren aged 6–7 years old. One year after implementing the program, the changes in the parents’ attitude and children’s behavior were marginally significant in the intervention group. According to the CAST index, there was less caries in deciduous molar teeth in the intervention group compared to the controls. In addition, a significant improvement observed in the oral hygiene status of children according to the OHI-S index in the intervention group compared to the control group. An improvement observed in the level of oral health related knowledge and self-reported behavior of parents that received the intervention package compared to the controls although the difference was not significant.

In the present study, evaluation of the caries status based on the CAST index showed that the percentage of caries in primary molars (CAST score ≥ 3) was significantly lower in the intervention group compared to the control group one year after the initial examination and receiving the educational interventions. Our findings are consistent with the results of longitudinal studies by Peterson et al., [20] and Tai et al., [21] that reported caries reduction based on the DMFT index in the intervention group receiving an educational package compared with the control group. However, they are in contrast to the results of a review study by Stein et al. in 2018 that did not find any long-term evidence regarding the effectiveness of educational interventions in caries prevention [22]. Moreover, in 2015, Jaime et al. reported that more subjects in the experimental group had higher knowledge of dental caries and declared that they used dental floss daily, but there was no significant difference in the incidence of dental caries between the case and control groups [23].

After one year, the children’s oral health status was compared between the control and intervention groups using the OHI-S index. In this regard, the findings of the present study showed a significant improvement in the oral health status of children in the intervention group in the one-year follow-up according to the OHI-S index. This finding was consistent with the results of a systematic review by Stein et al. in 2017 [22] that reported the impact of oral health education intervention on oral health. This meta-analysis found that the plaque index improved significantly based on the Loe and Silness index and O’Leary index following short-term educational interventions.

A recent review of Gargano et al., in 2019 advocated the involvement of parents in oral health programs in addition to stakeholders from different levels [24]. This is because children spend much time at home and therefore the active role of parents is important. In the present study, the oral health promotion program was effective in preventing caries progression in deciduous molar teeth and improving oral hygiene. This finding was in line with the results of a review study by Cooper et al. in 2013 [5] showing a statistically significant reduction in the plaque score in the test group receiving behavioral intervention compared to the control group. In addition, similar findings in two studies by Peterson et al. [20] and Jaime et al. [23] that used the Loe and Silness index for periodontal evaluation. Similarly, local studies conducted Saied-Moallemi et al. in 2009 [25] and Babaei et al in 2010 [17] reported the effectiveness of a school-based intervention.

In the present study, a comprehensive package including education workshop and supervised toothbrushing delivered. Our intervention package was prepared according to the recommendations of the World Health Organization (WHO) [1] including a training session for parents (mothers) and teachers in the form of a face-to-face workshop and pamphlet in addition to a supervised toothbrushing session for children in the school setting. A package including a toothbrush and fluoridated toothpaste was also given to children free of charge. Our finding was in line with a study by Peterson et al. in 2015 that reported that an oral health promotion training program including supervised toothbrushing with fluoride toothpaste was effective in reducing the incidence of caries [20]. Supervised toothbrushing is considered as an effective oral health promotion program for schoolchildren [5, 21]. However, a systematic review by Santos et al. in 2017 offered no conclusive recommendations regarding the effectiveness of supervised toothbrushing in the incidence of caries [26].

In the present study, both children and their parents were targeted for the oral health promotion program. According to the literature, the programs targeting children, parents, and teachers at the same time are more promising. This finding is consistent with a review study by Cooper et al. in 2013 that found that these interventions had a positive effect on maintaining oral health in children aged 4 to 12 years [5]. Similar to the studies conducted by Peterson et al. in 2015 [20] and Tai et al. in 2009 [21]. In this regard, Yekaninejad et al. conducted a local study in public elementary schools in 2012 and found that interventions targeting parents and school teachers in addition to children had more favorable results in promoting the children’s oral health [27].

After one year, the mean score of the parents’ oral health related knowledge, attitude and self-reported behavior was assessed in both groups. Although the parents’ attitude and the children’s behavior change were marginally significant in the intervention group, the difference was not significant. In this regard, the GEE analysis showed that the improvement of the parent’s attitude and children’s behaviors was marginally significant in the intervention group. In the present study, similar to a study by Gholami et al. [28], the GEE analysis was used to assess the impact of time and intervention considering the random sample effect to compare the intervention and control groups.

### Strengths

In the present study, a multi-stage cluster random sampling method was used, and a representative sample adjusted for the socio-economic status was recruited. To reduce the examiner bias, a previously validated questionnaire was used in addition to the CAST index to record the full spectrum of dental caries. Moreover, examiners in the present study were blinded and calibrated before data collection and used a visual aid containing CAST index classification details to reduce the risk of misclassification. In this trial, a comprehensive intervention package containing several components including supervised toothbrushing in school and a home package was used. For parents, the information delivered via a lecture, besides a pamphlet was provided for home use. As our sample was representative across the capital city of Tehran, local policymakers to improve the children’s oral health can use the results of our study.

### Limitations

This study had some limitations. In the process of data collection, some parents did not return the signed consent form and did not complete the questionnaire. However, reminders were sent to increase the response rate of the parents. Moreover, the process of collecting the completed questionnaires from parents was time-consuming. Besides, there was limited control over parents’ supervision of children’s brushing behavior at home. In order to overcome this limitation, a weekly plan (diary) was given to parents in order to facilitate supervision over their children. Furthermore, some parents were not interested in participation in the educational session. Since the CAST index is more complex compared to the DMFT/dmft index, the calibration and examination process required more time. However, in order to reduce the risk of misdiagnosis in the examination process, a visual aid containing the CAST index classification details was used during examination and calibration.

## Conclusions

Our study was conducted to evaluate the long-term impact of an oral health promotion intervention program including supervised toothbrushing and parents’ education on the oral health status of schoolchildren aged 6–7 years old. The children in the intervention group had less caries in deciduous molar teeth and a better oral hygiene status compared to children in the control group. In the intervention group, the changes in the parents’ attitude and the children’s behavior were marginally significant. The oral health related knowledge and self-reported behavior of parents in the intervention group did not improve significantly.

## Supporting information

S1 Checklist CONSORT 2010 checklist of information to include when reporting a randomised trial*. (PDF)

S1 File (SAV)
